# How medical professional students view older people with dementia: Implications for education and practice

**DOI:** 10.1371/journal.pone.0225329

**Published:** 2019-11-20

**Authors:** Theresa L. Scott, Melissa Kugelman, Kristen Tulloch

**Affiliations:** School of Psychology, The University of Queensland, St Lucia, Queensland, Australia; Fordham University, UNITED STATES

## Abstract

**Background:**

Healthcare professionals’ attitudes to older people, and especially those living with dementia, may contribute to unsatisfactory healthcare. Despite repeated calls to address increasing need, training an adequate geriatric workforce remains an international concern. Of particular concern are the attitudes and knowledge healthcare workers may hold about people living with dementia. Students’ knowledge of dementia has been found to be low at the beginning of their university education and has shown limited improvement throughout their coursework; greater understanding of students’ attitudes to ageing and dementia, upon entry and throughout their degrees, may help inform targeted educational interventions to improve the healthcare delivered to older people.

**Methods:**

This study measured knowledge of and attitudes toward dementia and ageing in an Australian university student sample (*n* = 183), comparing students from medical professional versus non-medical professional related fields at both undergraduate and postgraduate levels. We examined diagnostic and prognostic biases through age manipulation of a vignette describing a hypothetical patient (aged 42 or 72 years), who was experiencing symptoms that were consistent with DSM-5 criteria for both dementia and depression. Based on information provided in the vignette, student participants were asked to select a primary disorder that they would assign to the patient.

**Results:**

Showed that while medical professional students held significantly more positive attitudes toward ageing than 'other' students, average attitudinal scores indicated neutrality. Medical professional students indicated a diagnostic bias toward the older vignette patient, who was more likely to be diagnosed with dementia than depression. A history of geriatric-specific training did not predict dementia knowledge; however, having prior contact with people with dementia predicted both dementia knowledge and more positive prognoses.

**Conclusions:**

Overall, findings indicated medical professional students held neutral attitudes towards older people and showed deficits in knowledge of dementia. Educational interventions that introduce students to people living with dementia may improve knowledge, skills, and attitudes. All university students would benefit from education about dementia and inclusivity to reduce stereotyping and stigma.

## Introduction

There is a critical shortage of geriatrics-prepared healthcare professionals worldwide [1]. Australia, like most other regions of the globe, needs to prepare to meet the social and health care needs of a growing older adult population of unprecedented size, and substantial growth in numbers of people living with dementia. Dementia is a group of conditions characterised by cognitive decline, sometimes involving memory impairment, which affects an individual’s everyday functioning [2]. Dementia is a leading cause of death and disability around the world [3] and there are no curative measures. According to the World Health Organisation over 43.8 million people worldwide were living with dementia in 2016 [4]. Dementia is estimated to be increasing in prevalence at a faster rate than the total population, and population of older people in Australia [2, 5]. In Australia, three in 10 people aged 85 and over, and one in 10 people aged 65 and over, have dementia; consistent with global trends, these rates are increasing [6]. Young adults and student health professionals represent the next generation of carers; it is important to understand their views of mental health in late adulthood and their knowledge of dementia. Addressing age-related biases and misunderstandings about dementia through university curricula and training may be one way of impacting health equality for growing numbers of older people into the future.

Age is considered the most significant predictor of dementia and a high proportion of people living with a dementia are older adults [2]. Although dementia is now considered a group of diseases distinct from ageing [7], some clinicians and older adults still believe the misconception that dementia is a process of normal ageing [8, 9] due to stigma and low levels of dementia literacy [8]. Stigma and lack of knowledge can affect older adults’ health seeking behaviours, and the quality of healthcare that they receive [10]. Some studies have found health professionals to be inadequately trained in understanding, diagnosing, and caring for people with dementia, as recorded by both dementia knowledge measures and evaluations by patient families [11, 12]. Indeed, recent surveys in Europe, the United States, Asia and Australia have found knowledge about dementia was lacking in the general population [13]. For example, over half of 616 participants in one Australian survey study responded that ‘meaningful conversation was not possible’ with persons with dementia, and 34.0% said that they (people with dementia) were ‘irritating’. The majority (60%) said that if diagnosed themselves, they would feel a sense of shame [14]. Similar negative responses towards the progression of disease were found amongst adolescent students in one UK survey [15]. The majority of respondents agreed that the person with dementia will ‘eventually disappear’ (55.2%) and almost half (47.1%) agreed that ‘people with dementia were better off’ in residential care.

Sufficient knowledge of dementia is critical to ensuring better quality of care. Insufficient training might be responsible for misunderstandings and a lack of knowledge resulting in misdiagnoses, late diagnoses, and unsatisfactory care provided to people living with dementia [16–18]. A recent review of the literature pertaining to college students’ knowledge of dementia showed mixed results, in that some studies reported poor knowledge and others moderate or adequate knowledge, and some studies reported lower understanding of the risk factors and causes of dementia [19]. These mixed results were due, in part, to variability in sample, intervention, and measurement. For example, while medical students had higher scores on disease assessment, nursing students had more positive attitudes and preference for person-centred approaches than medical and pharmacy students. Unsurprisingly, increased time spent in dementia related coursework was associated with increased knowledge. Overall, the review showed that exposure to people living with dementia through supported, hands-on clinical experience resulted in improvement in students’ dementia knowledge and attitudes [19].

Age-related stereotypes held by health professionals and student health professionals include the belief that older people will have poor medical prognoses, are helpless, will eventually become ‘senile’, will not accept treatment recommendations, will have an unfulfilling later life, and don’t deserve healthcare [20–22]. These beliefs may manifest in discriminatory practices such as a failure to administer correct treatments, or any treatment at all, preferential treatment of younger patients, underdiagnosing depression and over-diagnosing dementia.

Dementia is a progressive neurodegenerative condition and the symptoms may be hard to distinguish from a depressive mood disorder. These shared symptoms can include impaired concentration, changes to cognition, memory and mood, and avolition [23]. Dementia is more common in older people and when cognitive symptoms are due to an affective disorder in older adults, health professionals can fail to recognise a potentially reversible cause [24]. Comorbidities are also common, with a recent meta-analysis suggesting up to 42% of individuals with dementia also experienced depression, depending on the population under consideration and the diagnostic criteria used [25]. Other common symptoms attributable to dementia may include anxiety, psychotic disturbances, and behavioural disturbances. Accordingly, diagnosing an individual’s condition requires careful examination of other presenting symptoms and the timeline of onset of features, among other considerations. Each of these may be subject to biases held by health professionals. Of particular concern is that under-diagnosis of depression and over-diagnosis of dementia is commonly reported in geriatric research, particularly when symptoms are present in an older adult [21, 22, 24]. Therefore, it may be necessary to address knowledge of, and attitudes toward dementia in undergraduate and pre-registration health programmes within the university curricula to meet the quality of care and quality of life needs of growing numbers of people living with dementia.

Medical professionals with some form of training or skill in elder care are more likely to provide appropriate care. For example, older patients cared for by nurses after geriatrics education and unit-based consultation were less likely to be physically restrained, compared to those whose nurses had no such preparation [26]. However, despite current training approaches, there is evidence that students remain underprepared to deliver the best care to older patients. A range of research has identified that nursing, psychology, and social work undergraduate and postgraduate students, along with postgraduate medical students have minimal knowledge of ageing and dementia at the beginning of their university study [27, 28], and that standalone geriatric coverage is not present in all undergraduate and postgraduate health discipline programs [29]. Furthermore, conflicting research has found that even when provided, university curricula inconsistently improve knowledge levels of students studying health-related degrees [30–32]. Research by Carpenter and colleagues [33] identified a 7-point difference in mean scores on the Alzheimer’s Disease Knowledge Scale (ADKS) between students of unreported discipline and degree level, and dementia health professionals, yet the true size of the knowledge gap between health students and health professionals remains to be seen. Comparisons in dementia knowledge between students of health versus other disciplines have not been made previously, and collection of such data may provide insight into the size of the knowledge gap demanding attention.

Care deficits in students and clinicians alike can be attributed in part to the presence of negative attitudes, biases and stereotypes held about older people [34–36]. These biases and stereotypes can be particularly damaging if they affect the quality of healthcare these older people receive, including disproportionate rates of misdiagnoses and delayed or missed treatment [16, 37]. Skinner, Scott and Martin [37] identified that forms of dementia were over-diagnosed in up to 50% of dementia cases in six studies, and that over-diagnosis was more common if there were more comorbidities or the disease was milder; however, one of their sources [38] appears to indicate that greater severity rather than lower severity returned more false positive errors. Skinner and colleagues also found insufficient sensitivity in studies, whereby dementia was under-diagnosed in up to 75% of cases, especially in those with risk factors including low socioeconomic status, lower education, and poorer access to healthcare. Although these results can seem contradictory, it is important to remember that these studies address two different populations: one that has the disease and one that does not; therefore, both over- and under-diagnosis can occur in the same study, given study samples draw on both populations and diagnosis is the process of determining to which population an individual belongs. Knowing an individual’s population membership is a challenge, and one which led Skinner and colleagues to call for investigation into factors that predispose misdiagnosis [37].

Considering the existing research, the present study explored the knowledge, attitudes and biases of an Australian undergraduate and postgraduate university student sample, comparing students enrolled in medical professional degrees with non-medical degree students. Students were asked about their knowledge of dementia risk factors, symptoms, life impact and management, and provided with a description of a patient with symptoms attributable to multiple psychological diagnoses. Based on this description, students were asked to select characteristics they felt described the patient, as a measure of their attitude towards the patient. Positive attitudes towards the patient included describing them as instrumental via terms such as strong, healthy or active; negative attitudes on the same scales included ineffective terms such as weak, unhealthy or passive. Using these conceptualisations, we predicted that:

- Students in medical professional-related degrees will have greater knowledge of dementia as shown by higher ADKS scores than those in non-medical degrees.
- The age of a patient will predict attitudes towards them as measured by the instrumental/ineffective dimension of the Ageing Semantic Differential (ASD), such that participants will be more likely to rate older patients more negatively than younger patients.
- Students will diagnose dementia more often than depression for the older patient than for the younger, and that this will be more pronounced in non-medical degree students.
- More contact with people living with dementia will predict greater dementia knowledge.
- Greater dementia knowledge will be related to fewer pessimistic health biases, such as their hope for the patient to have a fulfilling life, what the prognosis is likely to be, and how the patient will respond to treatment recommendations.
- Greater dementia knowledge will be related to more positive attitudes towards older people, shown by ratings consistent with the instrumental terms used in the ASD.

## Method

### Design

The study used a 2 (Degree: Medical Professional student or Other Student) x 2 (Vignette Patient Age: 42 or 72) between-subjects design. Participants were conservatively grouped according to their nominated course and career trajectories: i) students whose course led to a career with direct patient contact, such as medicine, nursing, dentistry, or ii) students in other courses (whose future career as a medical professional was indeterminate), such as business, engineering, media, arts, science, and undergraduate psychology. That is, in Australian programs, many students may take psychology courses as electives or complete an undergraduate degree and do not necessarily continue on to postgraduate training and clinical practice. We randomly assigned all participants to receive one of two vignettes that described a patient who was either 42 or 72 years old, for which further details are given below.

The outcome variables related to the vignette patient were attitudes towards the patient, health biases (i.e. ratings of the patient’s likelihood of living a happy and fulfilling life, openness to treatment recommendations, and prognosis), and primary diagnosis. Descriptive variables related to participants were contact with people living with dementia, knowledge of dementia, previous training experience, and interest in future geriatric training.

### Participant recruitment

Ethics approval for this survey study was granted by The University of Queensland Human Ethics Committee (number 17-PSYCH-4-52-AH). Current Australian university students were eligible for participation. We recruited medicine students from social media sites with which they engaged, and psychology students were recruited from the first-year student pool and given course credit for their participation. Other students were recruited via social media and snowball sampling methods.

### Participants

A total of 183 university students (70.5% female, 29.5% male) from 17 Australian universities were included in the analysis, aged 17 to 54 (*M =* 22.67, *SD* = 6.03). There were 77 students enrolled in medical professional degrees, aged 17 to 46 (*M* = 24.16, *SD* = 5.62), and 106 enrolled in non-medical professional degrees, aged 17 to 54 (*M* = 21.61, *SD* = 6.12). Overall, 90.7% of the sample were aged 30 years or younger. Participants who did not nominate a degree were excluded from analyses. As Australian tertiary courses can vary in duration for an undergraduate degree, specification of degree level is shown in Table 1, along with further demographic details.

**Table 1:** Demographics of participants.

	Total	Medical professional students	Other students
Number of participants by course	183	77	106
		Medicine/Biomedicine: 71	Science: 28
		Nursing & Paramedicine: 6	Psychology (undergraduate): 19
			Arts: 18
			Business: 7
			Engineering, Computer science & IT: 7
			Commerce, finance & economics: 5
			Education & Journalism: 5
			Law & Criminology: 4
			Health sciences: 5
			Other non-health: 8
Years of study			
First year	72	16	58
Second year	35	19	16
Third year	25	9	16
Fourth year	26	16	10
Fifth year	7	4	3
Sixth year	12	9	3
Seventh or higher year	4	4	0
Degree Level			
Diploma or certificate	3	0	3
Undergraduate	157	60	97
Postgraduate	23	17	6
Ethnicity*	Total	Health	Other
Caucasian	122	52	70
Australian Aboriginal / Torres Strait Islander	3	2	1
Pacific Islander	1	0	1
Black or African American	2	1	1
Middle Eastern	2	2	0
Asian	61	23	38
Other	1	1	0

* Students could enter multiple responses for ethnicity, so numbers of reported ethnicities do not equal the number of participants. One participant chose not to disclose their ethnicity.

### Procedure

A survey was administered to participants through the online software platform Qualtrics^™^. Participants were presented with demographic questions, a patient vignette (see Supporting Information 1), the Instrumental-Ineffective dimension of the Ageing Semantic Differential, the Alzheimer’s Disease Knowledge Scale and questions about their future career aspirations. We presented questionnaires in this order to avoid priming participants about dementia as a possible diagnosis.

#### Vignette

A vignette adapted from Helmes and Gee ([21]; Supporting Information 1) depicted a hypothetical patient aged either 42 or 72 years, randomised across participants. The patient, “Mr J”, was described as having symptoms that aligned with DSM-5 criteria for both depression and dementia; primarily avolition, behavioural and/or mood disturbance, and concentration difficulties [23]. Full DSM-5 diagnostic criteria are included as Supporting Information 2. As a manipulation check, participants were asked to rate how old they perceived the patient to be, on a scale of 1 (*young*) to 7 (*old*). This manipulation check was successful; a Spearman’s rank-order correlation confirmed that older patients were also perceived to be older, *r*_s_(181) = -.68, *p* < .001. Participants were instructed to provide a primary diagnosis for the patient described in the vignette by selecting from a list of 12 possible disorders including depression, dementia, substance-related disorder, anxiety or panic disorder, and bipolar disorder, among others. Multiple disorders were included to again avoid priming participants to dementia and/or depression. Although depression and dementia may be comorbid disorders, it was our intention to limit selection to one ‘primary’ diagnosis to educe potential biases.

#### Attitudes

The Instrumental-Ineffective dimension of the Ageing Semantic Differential (ASD; [39]) measured participants’ stereotypical attitudes towards the vignette patient. Ratings for the nine items are given on a 7-point Likert-type scale with anchors representing semantically opposite adjectives. For example, participants could be asked to rate a middle-aged person on a scale from 1 (*productive*) to 7 (*unproductive*). In the present study, students were asked to rate the vignette patient randomly assigned to them. Items included strong/weak, healthy/unhealthy, liberal/conservative and busy/idle. Item scores are summed with higher scores representing more negative stereotypical attitudes toward the vignette patient. The Instrumental-Ineffective dimension has factor loadings of .339 to .688 [40], and has shown acceptable reliability over four sequential cohorts of medical students (r = .77; [41]).

#### Knowledge

The Alzheimer’s Disease Knowledge Scale (ADKS; [27]) measured participants’ knowledge of dementia, specifically Alzheimer’s disease (AD). The ADKS consists of 30 statements that are considered myths or misconceptions about AD and participants label each item as either “true” or “false”. The statements are related to AD symptoms, treatments, risk factors, diagnosis and impacts, for example, “People with Alzheimer’s disease are particularly prone to depression”. Correct responses are summed to obtain an overall score, where higher scores indicate greater knowledge of AD. Preliminary psychometrics found that the ADKS yielded high internal consistency (α = .71 - .92) and was found to be a valid measure of AD knowledge in university students [33, 42].

#### Health biases

Three separate items were used to measure health biases. Participants rated the hypothetical patient’s (i) likelihood of living a happy and fulfilling life, (ii) openness to treatment recommendations and (iii) prognosis. These biases were based on those identified by Helmes and Gee [21], and James and Haley [43]. Ratings were made on a 7-point Likert-type scale for all measures. For example, in anticipating the patient’s prognosis, participants rated on a scale from 1 (*poor*) to 7 (*excellent*). Higher scores on each measure represented more optimistic health biases.

#### Contact, interest, and training measures

In addition, participants’ contact with older people, prior training experience, and future interest in working with older people, were all binary measures, with responses coded as either *yes* or *no*. Contact with people with dementia was measured by responses to the item “Do you know anyone (not including yourself) with dementia?” Item responses included (i) family member, (ii) friend, (iii) acquaintance, (iv) specified other, (v) and no; recoded as a binary ‘yes’ or ‘no’ variable. Table 2 shows the frequency of the categorical variables across each student group and the student sample overall.

**Table 2:** Frequencies of focal categorical variables for total sample and breakdown by student group.

	% Yes responses		
Categorical Variable	Total overall	% Medical degree	% Other degree
Do you have any significant experience in caring for older adults (e.g. work or family role)?	23.5	27.3	20.8
Have you had any training or prior experience working with older adults?	19.9	32.5	10.6
Do you anticipate working with older adults in your career?	52.5	76.6	52.5
Do you know anyone (not including yourself) with dementia?	45.4	59.7	50.9

#### Data analysis

Statistical Package for the Social Sciences (SPSS) Version 22 [44] was used for quantitative data analysis. Descriptive statistics were used to summarize sociodemographic data and participants’ previous training and experiences, professional aspirations, and contact with older adults and people living with dementia.

## Results

Means and standard deviations for continuous variables of dementia knowledge (ADKS), attitude to patient (ASD), measures of health biases (happy life, openness to treatment, prognosis) and training measures (own interest in geriatric studies) and *t*-tests of significance between the student groups, are shown in Table 3.

**Table 3:** Means (*M*), Standard Deviations (*SD*) and ranges of focal continuous variables.

	Total			Medical professional degrees			Other degrees		
Variable	*M*	*SD*	Range	*M*	*SD*	Range	*M*	*SD*	Range
ADKS scores	22.44	3.96	12–30	24.41***	3.31	15–30	21.05	3.80	12–30
ASD composite score	4.60	0.57	3.22–6.33	4.48*	0.52	3.33–5.89	4.69	0.59	3.22–6.33
Happy life ratings	4.44	1.41	2–7	4.16*	1.57	2–7	4.64	1.26	2–7
Openness to treatment ratings	3.84	1.26	1–7	3.61*	1.27	1–6	4.01	1.23	1–7
Prognosis ratings	4.14	1.13	1–7	3.92*	1.11	2–7	4.30	1.13	1–7
Interest in geriatric study	4.06	1.67	1–7	4.34	1.50	1–7	3.86	1.76	1–7

Significance values for *t*-tests

*p < .05, ** *p* < .01

***p < .001. ADKS scores range 30 = 100% correct; ASD composite higher scores = more negative stereotypical attitudes. Scaled responses: 1 = (*not at all interested*) to 7 (*very interested*).

### Attitudes toward ageing

**Fig 1:**
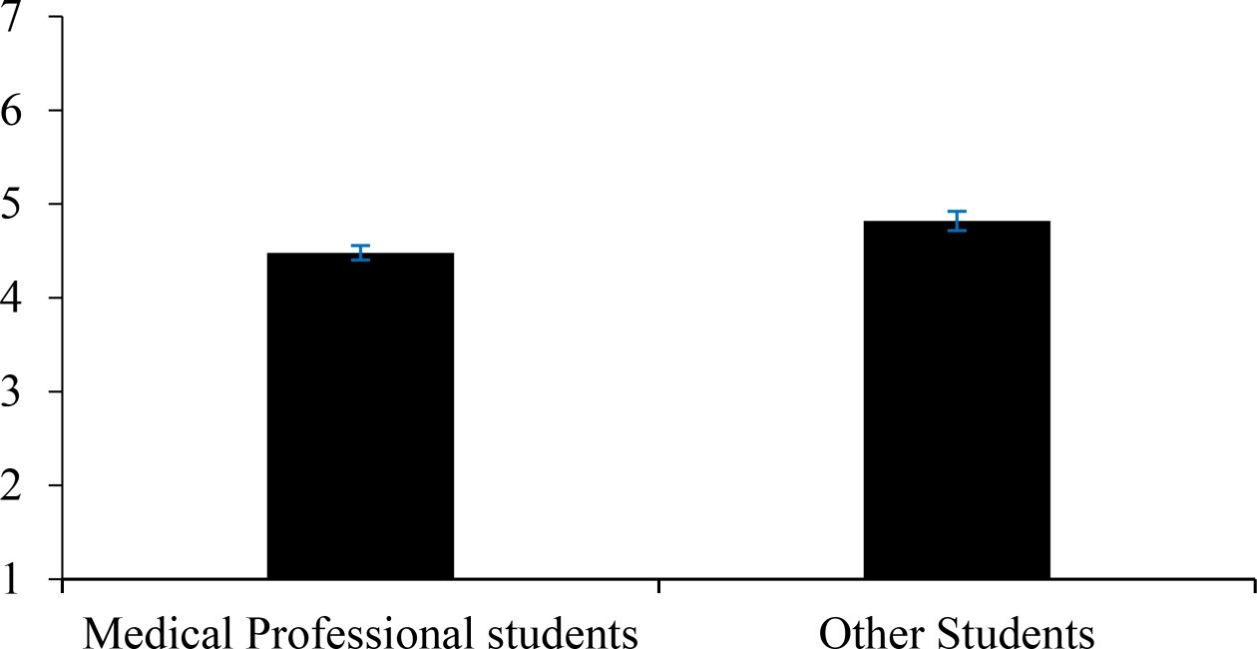
Mean scores of attitudes towards the older patient for each student group.

We examined differences in attitudes toward ageing, ASD scores, according to student groups. Firstly, a *t*-test indicated that attitudes towards the older versus younger patients were not significantly different, *t*(181) = 1.66, *p* = .098. However, when comparing the data for each patient age, a linear regression identified that degree significantly explained 8.3% of attitudes towards the older patient, *F*(1, 83) = 7.55, *p* = .007. Average attitudes towards the older patient were significantly more positive for medical professional students than other students, however still around the mid-point as shown in Fig 1, *t*(83) = 2.75, *p* = .007.

Next, we examined whether participants’ attitudes toward the vignette patient differed according to degree category and patient age, a 2 (student group: health and other) X 2 (vignette patient: 42 and 72 years) ANOVA was conducted. Results showed a significant main effect of student group only. That is, students in ‘other’ degrees endorsed significantly more negative statements overall than students in health degrees, as noted above.

A focal bivariate correlation was conducted with knowledge and attitudes and revealed that the variables were significantly related, *r*(181) = -.24, *p* = .001. This indicated that participants who were more knowledgeable had more negative attitudes towards the patient.

### Knowledge of dementia

As shown in Table 3, medical professional students’ knowledge of dementia as measured by their scores on the ADKS was significantly higher than that of other students *t*(181) = 6.22, *p* < .001). A linear regression was conducted with degree as a predictor of knowledge of dementia. The analysis revealed that degree significantly explained 17.6% of variance in knowledge scores, *F*(1, 181) = 38.65, *p* < .001.

A multiple linear regression was performed with training experience and contact with people with dementia as predictors, and knowledge as the outcome variable. Training experience did not significantly predict variance in knowledge scores. However, contact with people with dementia was a significant predictor of knowledge. Overall, the model significantly accounted for 6.1% of the variance, *F*(2, 180) = 5.86, *p* = .003. Regression coefficients and standard errors can be found in Table 4.

**Table 4:** Beta weights for regression analyses.

Variable	*Unstandardized B*	*SE*_*B*_	β
Intercept	21.26	0.45	
Contact with people living with dementia	1.53	0.58	0.19
Have you had prior training or experience working with older adults	1.19	0.64	0.14

### Diagnostic bias

**Fig 2:**
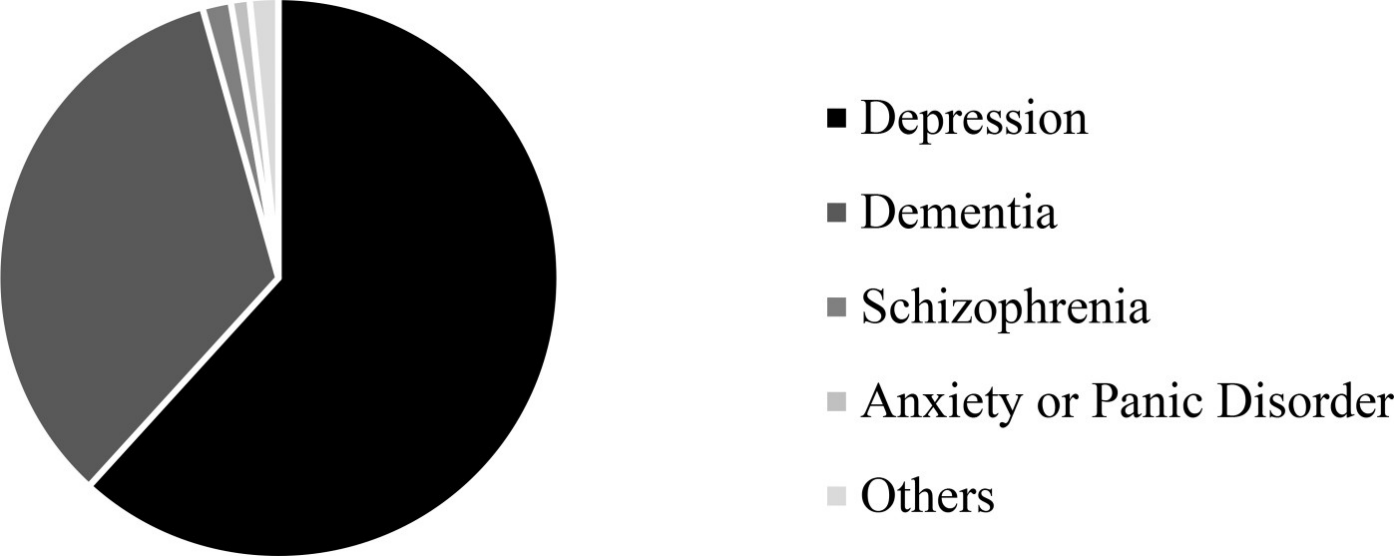
Frequency of each primary diagnosis selected for patients by all participants.

While 12 possible diagnostic options were provided to participants, most selected a preliminary diagnosis of depression or dementia, as shown in Fig 2. The following analyses specifically explored a diagnostic bias relating to depression and dementia diagnoses, for which we created a new variable where 0 = depression diagnosis, and 1 = dementia diagnosis. Since all other diagnoses represented less than 5% of all responses, these participants were included in the following analyses as missing data. To investigate the presence of a diagnostic bias, chi-square tests of independence and a logistic regression analysis were conducted.

**Fig 3:**
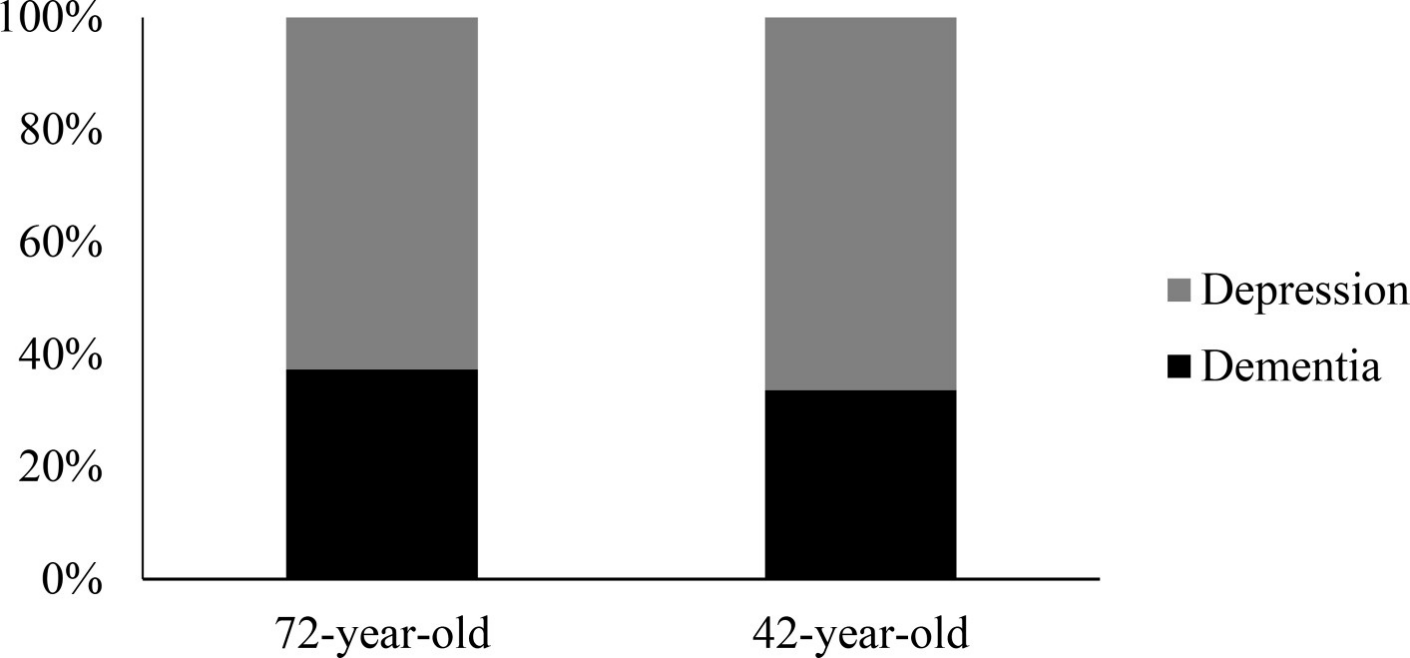
Percentage of dementia and depression diagnoses selected by participants for each vignette patient.

The chi-square test found no significant relationship between diagnosis and patient age, χ^2^(1, *N* = 175) = 0.26, *p* = .614. This indicated that diagnoses were not contingent on the age of the patient. However, participants gave a higher percentage of dementia diagnoses for the 72-year-old patient compared to the 42-year-old patient. Specifically, dementia represented 37.3% of all diagnoses given to the 72-year-old patient, and 33.7% of all diagnoses given to the 42-year-old patient, as shown in Fig 3.

A logistic regression was performed with diagnosis as the outcome and the five predictors of knowledge, attitudes, happy life, openness to treatment, and prognosis. A total of 85 cases were analysed and attitudes, all health biases, and the full model for the 72-year-old patient were not statistically significant, χ^2^(5, *N* = 85) = 6.68, *p* = .246. However, knowledge was a significant predictor of diagnosis for the 72-year-old patient, such that an increase in knowledge scores by 1 indicated a decrease in odds of a dementia diagnosis by a factor of 0.88, 95% CIs [0.79, 0.99].

The full model for the 42-year-old patient was statistically significant, χ^2^(5, *N* = 98) = 14.91, *p* = .011. The model explained between 15% and 20.7% of variance in the data; however, attitudes and all three health biases were not significant predictors. Knowledge was a significant predictor of diagnosis, such that an increase in knowledge scores by 1 was associated with a decrease in odds of a dementia diagnosis by a factor of 0.77, 95% CIs [0.66, 0.90]. Table 5 provides coefficients and Wald statistics for all predictors in this analysis.

**Table 5:** Strength of predictors and change in odds (Exp B) of a depression diagnosis of each predictor for each patient.

	72-year-old		42-year-old	
Variable	Wald	Exp(B)	Wald	Exp(B)
ADKS	4.48*	0.88	10.71*	0.77
Attitudes	0.39	0.79	0.50	1.38
Happy life	1.88	0.73	0.18	0.92
Openness to Treatment	0.02	1.03	0.98	0.83
Prognosis	0.69	1.27	0.16	1.12

*p < .05.

**Fig 4:**
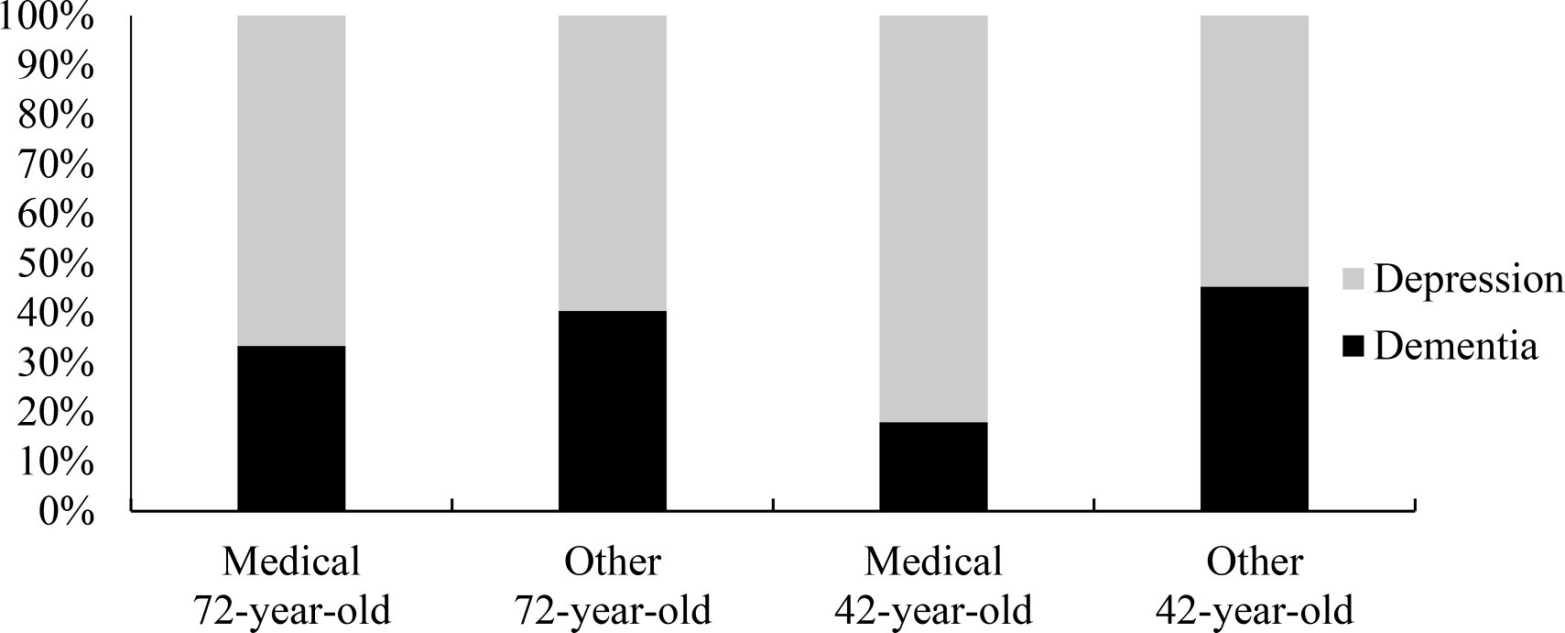
Percentage of dementia and depression diagnoses selected by each student type for each vignette patient.

A chi-square test of independence was conducted using degree and diagnosis however Yates’ correction for continuity was used for significance since small cell counts violated assumptions of the Pearson chi-square statistic [45]. A significant relationship was found between the variables for participants in the 42-year-old patient condition, χ^2^(1, *N* = 92) = 6.34, *p* = .012. This effect was not evident for participants in the 72-year-old patient condition, χ^2^(1, *N* = 83) = 0.19, *p* = .665. Of the students in medical professional degrees assessing a 42-year-old patient, 82.1% diagnosed their patient with depression, and 17.9% diagnosed their patient with dementia. In comparison, other students gave almost equal proportions of each diagnosis to the 42-year-old patient, as shown in Fig 4. This suggests greater diagnostic bias in medical degree students than non-medical degree students.

## Discussion

This research explored the attitudes, knowledge and biases of two cohorts of Australian university students; those enrolled in medical professional degrees compared to those enrolled in non-medical professional degrees. Students were presented with ambiguous information about a fictional patient and asked to provide a primary diagnosis, then to answer questions about their perceptions of the patient, details of their enrolment, and their expectations for their future involvement with older clients as health practitioners. This research provides some insight into students’ perceptions of, and attitudes towards older people as patients. Limited dementia literacy, and negative ideations toward older people and people living with dementia have significant detrimental effects on quality of life and equality of healthcare provided to older people.

Initially, we predicted that medical professional students would have greater knowledge about dementia than non-medical professional students. This hypothesis was supported, and is an interesting finding given we also found that medical professional students in the present study did not have significantly greater contact or training with people with dementia than the non-medical professional students. The reason for this discrepancy is beyond the scope of the present study but may be a result of students’ self-selection of their degrees, a higher proportion of postgraduate students within the medical compared to non-medical samples, or a higher proportion of more advanced students in the medical sample, as many enter the first year of medicine having completed another bachelor degree. The dementia knowledge of students of unknown disciplines reported by Carpenter and colleagues [33] was lower than that of our health students, but higher than our non-health students. Prior research found that both clinicians and older adults hold the misconceptions that dementia is a process of normal ageing [8,9], however it would seem that our sample of medical professional students have improved understandings in terms of overall knowledge of dementia. For example, mean score of the ADKS for health professional students indicates an overall accuracy level of 81%.

We further predicted that students would hold more negative attitudes toward the older patients; this hypothesis was not supported. Medical professional students held significantly more positive attitudes to the older patient than non-medical professional students. However, it is important to note that the average attitudinal scores were around the mid-point of the scale, indicating that both groups held relatively neutral attitudes towards older people. This neutrality is consistent with previous research, where Stewart and colleagues [41] found students scored 70% of items neutrally. Stewart and colleagues suggested neutrality in their study could have been a function of genuinely neutral attitudes, low relatability of the adjectives used in the questionnaire which prompted more neutral responses for students, or the reduced response range Stewart and colleagues offered, of 1–5 rather than 1–7. In the present study, the vignette describing poor health may have introduced additional bias. Gekoski and Knox [46] conducted a study that identified that only individuals who were in “poor health” were rated negatively using the ASD. The researchers posited that the presence of “ageism” in the health system may be the product of the poor health that covaries with age, a phenomenon known as “healthism”. While Gekoski and Knox’s research presents a possible explanation for the findings of the current study, it is unclear why these health biases were only identified among students in health-related degrees for younger patients. Thus, future research should further explore the phenomenon of healthism among those involved in health professions and society in general to explore whether it accounts for ageist attitudes, or negative beliefs in general.

We also predicted that students would diagnose the older patient with dementia significantly more than the younger patient, and that this would be more pronounced in non-medical professional students. This hypothesis was not supported; primary diagnoses of depression and dementia were provided at similar rates to older and younger patients, except for the 42-year-old diagnosed by medical professional students, who were more likely than non-medical professional students to diagnose depression for that individual. This difference may be attributable in part to differences between the two student groups and within the medical professional group. That is, medical professional students are exposed to more depression related course content than non-medical students, and to more depression content than dementia content. Furthermore, depression related information that is embedded in resilience programs targeted at medical professional students [47] may result in increased awareness of depression and the likelihood of an assessment of depression for the younger patient compared to the older patient. Our finding is inconsistent with literature that identified higher rates of dementia diagnoses for older people [18, 48, 49], however no previous study has empirically assessed the presence of a diagnostic bias using a manipulation like that of the current study. Thus, these results should be assessed further in research to greater understand the origin of such diagnostic biases.

Our hypothesis that previous contact with people living with dementia would result in greater dementia knowledge was supported, consistent with existing literature [45, 46]. Increased knowledge of dementia that arises from contact with people living with dementia indicates a possible method for ensuring greater knowledge levels among both current and students in medical professional degrees. Given the benefits demonstrated in this study whereby greater knowledge of dementia relates to fewer biases, along with more person-centred attitudes shown in previous research [50], future research should explore the ways in which contact benefits can be achieved for future health workers.

Similarly, our hypotheses that greater dementia knowledge would relate to fewer biases and more positive attitudes was partially supported, that is, greater knowledge was associated with more negative attitudes, which was only partially consistent with previous literature [18, 31, 32]. However, this result may represent the tendency for all participants to select a depression diagnosis over one of dementia due to its prevalence or salience in society. This has implications for the promotion of community education and awareness raising to promote inclusive dementia friendly communities, much the same way as campaigns that have increased awareness of mental health and in this case depression.

Given the nature of the research conducted here, it is not possible to determine causality in the relationships observed. However, these findings provide an interesting basis for future research into dementia-related and medical professional education. This research could create insight about the effectiveness of university education as a means of training students in medical professional degrees and provide a comparative understanding of what other university students know and believe.

### Implications

Participants who had had contact with individuals with dementia were more knowledgeable. Thus, a practical implication of the current study is that greater exposure to people with dementia could improve all students’ understanding of the risks, symptoms, and course of the diseases. This finding could be used in designing training for students in medical professional-related degrees to facilitate improvement in their knowledge levels. For example, guest tutorials and lectures from people advocating for and living with early- and late-onset dementias could improve students’ knowledge while potentially preventing biases from forming. Postgraduate students could be offered more in-depth clinical training with a range of people living with dementia, such as those newly diagnosed and living in the community and those with more advanced dementias who are living in care facilities.

Another implication of these findings is the need for a potential amendment of current university training, both for students in medical professional degrees and other students. Filling knowledge gaps, improving attitudes towards, and removing stereotypes about older adults would be beneficial to all university students considering the observed deficits. Furthermore, if this was implemented as explicit training for all students such as via clinical contact [19], the healthcare system and general workforce would be better prepared to cater for the growing proportion of older adults in Australia [51–53].

### Strengths, limitations and future directions

This study is novel in that it explores the attitudes and knowledge of dementia of a sample of university students whose future career choices include medical and non-medical related professions. Medical professional students showed greater dementia knowledge than students from non-medical related disciplines. However, one-third of the sample of medical professional students do not see themselves working with older adults in their future careers, this is a concerning finding given that older people represent a significant and growing proportion of consumers of health care.

In comparing student groups, students in medical professional degrees gave more depression diagnoses to younger patients. Contact with people with dementia was related to increased dementia knowledge, which was in turn related to fewer biases, demonstrating the importance of increasing dementia knowledge in both future clinicians and the wider community. Nevertheless, this study is unable to comment on the causality of relationships shown here. Furthermore, the results should be interpreted in line with the limitations of the cross-sectional nature of the student sample. That is, attitudes and knowledge scores were averaged across student groups in which first year students were included with more advanced students. Future studies which use a longitudinal design would be informative here. It is further advisable that these findings be considered in the context of dementia prevalence. It needs to be acknowledged that unlike in professional practice, students were not able to give a differential diagnosis. However, while age is a strong predictor of dementia, the results seem to suggest that age-related biases remain and highlight a need for new clinicians to be self-aware of age-related stereotypes and the potential for aged-biased clinical decision-making.

### Conclusions

In sum, the present study demonstrated the knowledge, attitudes and biases of students in medical professional degrees and other university students regarding older adults and dementia. These findings expand the understanding of current training standards of future clinicians and non-medical professionals by identifying deficits in knowledge, possible negative attitudes, and biases regarding older people. This research has practical implications for the current university curriculum and training of all university students in preparing them for future work in an ageing world.

## Supporting information

S1 File Patient vignettes. Vignettes describing two patients; one aged 42 years and one aged 72 years.
(DOCX)

S2 File DSM-5 diagnostic criteria. Diagnostic Criteria for Mild and Major Neurocognitive Disorder and Major Depressive Disorder.
(DOCX)
