# Supplementary material for: How medical professional students view older people with dementia: Implications for education and practice
Source: PLoS One. 2019 Nov 20;14(11):e0225329. doi: 10.1371/journal.pone.0225329 (PMC6867636; doi:10.1371/journal.pone.0225329)
Supplement: S1 File — Vignettes describing two patients; one aged 42 years and one aged 72 years. (DOCX) [file pone.0225329.s001.docx]

Supporting Information 1: Original Vignette and Restrictions Items Prior to Pilot Testing

Mr. J. is a **72/42**-year-old man who has been a long-term patient at your medical centre. He books an appointment with you and arrives with his wife and you note that he appears apathetic. He explains that he is no longer interested in hobbies he used to love like playing guitar and watching cricket games. His wife explains that he hardly leaves the house and has isolated himself from a lot of his friends. During your consultation, you notice that he seems to have trouble concentrating on your conversation and struggles when coming up with answers to your questions.

Based on your chosen diagnosis, do you think that this will affect their activities of daily living and ability to work, socialise etc.? If so, what restrictions or recommendations might you as a physician impose for this patient?
