# Supplementary material for: How medical professional students view older people with dementia: Implications for education and practice
Source: PLoS One. 2019 Nov 20;14(11):e0225329. doi: 10.1371/journal.pone.0225329 (PMC6867636; doi:10.1371/journal.pone.0225329)
Supplement: S2 File — Diagnostic Criteria for Mild and Major Neurocognitive Disorder and Major Depressive Disorder. (DOCX) [file pone.0225329.s002.docx]

**Supporting Information 2:**

**DSM-5 Diagnostic Criteria for Mild and Major Neurocognitive Disorder and Major Depressive Disorder**

**American Psychiatric Association, 2013**

### Mild Neurocognitive Disorder

1. Evidence of modest cognitive decline from a previous level of performance in one or more cognitive domains (complex attention, executive function, learning and memory, language, perceptual-motor, or social cognition) based on:
   1. Concern of the individual, a knowledgeable informant, or the clinician that there has been a mild decline in cognitive function; and
   2. A modest impairment in cognitive performance, preferably documented by standardized neuropsychological testing or, in its absence, another quantified clinical assessment.
2. The cognitive deficits do not interfere with capacity for independence in everyday activities (i.e., complex instrumental activities of daily living such as paying bills or managing medications are preserved, but greater effort, compensatory strategies, or accommodation may be required).
3. The cognitive deficits do not occur exclusively in the context of a delirium.
4. The cognitive deficits are not better explained by another mental disorder (e.g., major depressive disorder, schizophrenia).

### Major Neurocognitive Disorder

1. Evidence of significant cognitive decline from a previous level of performance in one or more cognitive domains (complex attention, executive function, learning and memory, language, perceptual-motor, or social cognition) based on:
   1. Concern of the individual, a knowledgeable informant, or the clinician that there has been a significant decline in cognitive function; and
   2. A substantial impairment in cognitive performance, preferably documented by standardized neuropsychological testing or, in its absence, another quantified clinical assessment.
2. The cognitive deficits interfere with independence in everyday activities (i.e., at a minimum, requiring assistance with complex instrumental activities of daily living such as paying bills or managing medications).
3. The cognitive deficits do not occur exclusively in the context of a delirium.
4. The cognitive deficits are not better explained by another mental disorder (e.g., major depressive disorder, schizophrenia).

**Major Depressive Disorder**

1. Five (or more) of the following symptoms have been present during the same 2-week period and represent a change from previous functioning; at least one of the symptoms is either (1) depressed mood or (2) loss of interest or pleasure.
   - **Note:** Do not include symptoms that are clearly attributable to another medical condition.
   1. Depressed mood most of the day, nearly every day, as indicated by either subjective report (e.g., feels sad, empty, hopeless) or observation made by others (e.g., appears tearful). (**Note:** In children and adolescents, can be irritable mood.)
   2. Markedly diminished interest or pleasure in all, or almost all, activities most of the day, nearly every day (as indicated by either subjective account or observation).
   3. Significant weight loss when not dieting or weight gain (e.g., a change of more than 5% of body weight in a month), or decrease or increase in appetite nearly every day. (**Note:** In children, consider failure to make expected weight gain.)
   4. Insomnia or hypersomnia nearly every day.
   5. Psychomotor agitation or retardation nearly every day (observable by others, not merely subjective feelings of restlessness or being slowed down).
   6. Fatigue or loss of energy nearly every day.
   7. Feelings of worthlessness or excessive or inappropriate guilt (which may be delusional) nearly every day (not merely self-reproach or guilt about being sick).
   8. Diminished ability to think or concentrate, or indecisiveness, nearly every day (either by subjective account or as observed by others).
   9. Recurrent thoughts of death (not just fear of dying), recurrent suicidal ideation without a specific plan, or a suicide attempt or a specific plan for committing suicide.
2. The symptoms cause clinically significant distress or impairment in social, occupational, or other important areas of functioning.
3. The episode is not attributable to the physiological effects of a substance or another medical condition.
4. The occurrence of the major depressive episode is not better explained by schizoaffective disorder, schizophrenia, schizophreniform disorder, delusional disorder, or other specified and unspecified schizophrenia spectrum and other psychotic disorders.
5. There has never been a manic episode or a hypomanic episode.
6. **Note:** This exclusion does not apply if all of the manic-like or hypomanic-like episodes are substance-induced or are attributable to the physiological effects of another medical condition.
